# Serum mir-181a as a potential serological biomarker for metabolic dysfunction-associated fatty liver disease: A cross sectional study

**DOI:** 10.1097/MD.0000000000047290

**Published:** 2026-01-23

**Authors:** Ke-Gong Xiong, Jin-Feng Kong, Tai-Shun Lin, Qing-Biao Lin, Kun-Yu Ke

**Affiliations:** a Department of Hepatology, Mengchao Hepatobiliary Hospital of Fujian Medical University, Fuzhou, China.

**Keywords:** 181a, associated fatty liver disease, diagnostic biomarker, metabolic dysfunction, miR

## Abstract

This study aimed to investigate the expression and clinical significance of serum microRNA-181a (miR-181a) in patients with metabolic dysfunction-associated fatty liver disease (MAFLD). In this cross sectional study, we enrolled patients diagnosed with MAFLD and non-MAFLD individuals undergoing routine physical examinations as controls between January 2023 and December 2024. Serum miR-181a levels were quantified using real time quantitative polymerase chain reaction (RT-qPCR). Serum miR-181a levels were significantly higher in the MAFLD group compared to those in the control group (1.15 ± 0.50 vs 0.79 ± 0.39, *P* < .001). MiR-181a expression is significantly associated with adverse metabolic parameters, liver injury, and fibrosis. Elevated miR-181a expression was identified as an independent risk factor for MAFLD (OR = 2.295, 95% CI: 1.318–6.236, *P* = .011). The optimal serum miR-181a cutoff value for diagnosing MAFLD was determined to be 0.90, resulting in an area under the curve of 0.80, with a sensitivity of 77.50% and a specificity of 76.67%. MiR-181a is significantly associated with the pathogenesis and progression of MAFLD and may serve as a promising serological biomarker for diagnosis.

## 1. Introduction

Metabolic dysfunction-associated fatty liver disease (MAFLD), a term introduced in 2020 to replace nonalcoholic fatty liver disease, is a chronic and progressive liver disorder that originates from metabolic dysregulation (MD).^[1]^ It was further updated and revised in 2025.^[2]^ Driven by the rising global prevalence of obesity, type 2 diabetes mellitus (T2DM), and metabolic syndrome, MAFLD has emerged as the most common chronic liver disease worldwide, affecting over 25% of the population.^[2,3]^ This condition poses a significant threat to human health due to its potential progression to liver fibrosis, cirrhosis, and hepatocellular carcinoma. The MAFLD nomenclature underscores the critical role of underlying metabolic dysfunction in disease pathogenesis and facilitates improved patient classification and management. Currently, the absence of specific and reliable serological diagnostic markers continues to represent a significant challenge in the clinical management of MAFLD.

MicroRNAs (miRNAs) are small noncoding RNA molecules that play a crucial role in regulating gene expression, thereby influencing cellular phenotypes and functions. Their dysregulated expression has been observed in various diseases, making them promising candidates for biomarker development.^[4]^ microRNA-181a (MiR-181a), a key member of the miR-181 family and a noncoding small RNA, serves as a critical regulator in diverse biological processes. Emerging evidence positions miR-181a as a key regulator in metabolic disorders, primarily through its disruptive impact on lipid metabolism. Its overexpression in hepatocytes suppresses the Sirt1/PGC-1α axis, which exacerbates hepatic steatosis and disrupts glucose homeostasis by promoting insulin resistance and reducing glycogen storage.^[5]^ A central mechanism for this lipid accumulation is the miR-181a-mediated downregulation of PPARα, a master regulator of fatty acid oxidation.^[6]^ Furthermore, beyond MD, miR-181a exhibits profibrotic properties by activating pro-inflammatory pathways and directly stimulating hepatic stellate cells, thereby contributing to liver fibrosis.^[7]^

However, the clinical translation of these mechanistic insights remains limited, as the expression profile and diagnostic significance of circulating miR-181a in MAFLD patients are insufficiently understood. Therefore, we hypothesized that serum miR-181a levels are elevated in MAFLD and associated with disease severity, representing a promising noninvasive biomarker for this condition.

## 2. Methods

### 2.1. Study population

This observational cross sectional study was conducted at Mengchao Hepatobiliary Hospital of Fujian Medical University between January 2023 and December 2024. Sample size was determined a priori using PASS 2021 for receiver operating characteristic (ROC) curve analysis. Assuming an area under the curve of 0.75, α = 0.05, and 90% power, 108 participants were required. Therefore, we enrolled 180 participants (120 MAFLD patients and 60 controls) to support a 2:1 enrollment ratio and ensure robust subgroup analyses.

The diagnostic criteria for MAFLD follow the Asian Pacific Association for the study of the liver clinical practice guidelines for the diagnosis and management of MAFLD.^[2]^ This criterion requires evidence of liver fat accumulation plus at least one of the following: body mass index (BMI) ≥ 23 kg/m², T2DM, or MD, which includes increased waist circumference, hypertension, hyperlipidemia, hyperglycemia, and insulin resistance. The control group was defined as individuals from the same time period who underwent routine health checkups and showed no imaging evidence of hepatic steatosis. Exclusion criteria for both groups included chronic viral hepatitis, other established liver diseases (e.g., autoimmune or drug-induced), significant alcohol consumption, parasitic infections, malignancies, and severe systemic conditions. The study received ethics approval from Fujian Medical University Mengchao Hepatobiliary Hospital (No. 2023–095-01) and complied with the Declaration of Helsinki. All participants provided written informed consent prior to enrollment.

### 2.2. Sample and data collection

All subjects underwent fasting venipuncture (5 mL blood collected from the antecubital vein), and samples were placed in EDTA anticoagulant tubes. The blood was centrifuged at 3000 rpm for 10 minutes, after which the serum was separated and stored at −80°C. Clinical data, including age, gender, BMI, T2DM, hypertension, albumin (ALB), total bilirubin (TBIL), alanine aminotransferase (ALT), aspartate aminotransferase (AST), gamma-glutamyl transferase (GGT), alkaline phosphatase (ALP), total cholesterol (TC), high-density lipoprotein cholesterol (HDL-C), low-density lipoprotein cholesterol (LDL-C), triglycerides (TG), fasting plasma glucose, uric acid (UA), HbA1c, high-sensitivity C-reactive protein (hs-CRP), fibrosis-4 index (FIB-4), and liver stiffness measurement (LSM) were extracted from the hospital’s electronic medical record system. FIB-4 was calculated as (age × AST) divided by (PLT count × square root of ALT).

### 2.3. Detection of serum miR-181a

Serum miR-181a expression levels were quantified using real time quantitative polymerase chain reaction (RT-qPCR). Total RNA was extracted from 200 μL of serum samples using an RNA extraction kit. The RNA was reverse transcribed into complementary DNA (cDNA) using a reverse transcription kit. The resulting cDNA was used as a template for qPCR amplification on a real time fluorescence quantitative PCR system. The qPCR reaction program included an initial activation step at 95°C for 10 minutes, followed by 40 cycles of denaturation at 95°C for 15 seconds, annealing at 55°C for 30 seconds, and extension at 72°C for 30 seconds. Specific primers targeting miR-181a and the reference gene GAPDH (Wuhan Savel Biotechnology Co., Ltd., Wuhan, China) were designed and purchased. MiR-181a expression levels were calculated using the 2−^ΔΔCt^ method and normalized to GAPDH.

### 2.4. Statistical analysis

Statistical analyses were performed using SPSS version 23.0 (SPSS Inc., Chicago). Continuous variables were expressed as mean ± standard deviation, and the independent samples *t*-test was applied for intergroup comparisons, while 1-way analysis of variance was used for comparisons among multiple groups. Categorical variables were presented as percentages (%) and analyzed using chi-square (χ²) tests. Stratified analysis was performed to explore the influence of key metabolic features on miR-181a expression in MAFLD patients. The cohort was stratified according to 3 criteria: BMI (lean [<23 kg/m²] vs non-lean [≥23 kg/m²]), T2DM status, and the presence of MD. Spearman correlation analysis was conducted to assess the association between miR-181a expression and metabolic, liver function, and fibrosis indicators. Logistic regression analysis was utilized to identify risk factors associated with MAFLD. The diagnostic value of miR-181a in detecting MAFLD was assessed using ROC curve analysis. A 2-tailed *P*-value < .05 was considered statistically significant.

## 3. Results

### 3.1. Baseline characteristics of this study cohort

The prevalence of MAFLD patients with BMI ≥ 23 (kg/m²), T2DM, and MD was significantly higher than that in the control group (all *P* < .05). Patients in the MAFLD group exhibited elevated levels of BMI, ALT, AST, GGT, ALP, TG, TC, LDL-C, fasting blood glucose, UA, FIB-4, and LSM compared to the control group (all *P* < .05). In contrast, HDL-C levels were significantly lower in the MAFLD group (*P *= .002). No statistically significant differences were observed in age, sex distribution, ALB, or TBIL between the 2 groups (all *P* > .05) (Table 1).

**Table 1:** Baseline characteristics of this study cohort.

Variables	MAFLD (n = 120)	Control group (n = 60)	*P* value
Age (yr)	42.08 ± 12.20	42.12 ± 12.68	.983
Male	79 (65.8%)	34 (56.7%)	.230
BMI (kg/m^2^)	25.57 ± 2.41	22.70 ± 2.70	<.001
BMI ≥ 23 (kg/m^2^)	103 (85.8%)	35 (58.3%)	<.001
T2DM	21 (17.8%)	4 (6.7%)	.048
MD	34 (28.3%)	8 (13.3%)	.025
ALB (g/L)	47.35 ± 2.57	46.69 ± 2.43	.092
TBIL (µmol/L)	13.30 ± 5.47	13.13 ± 3.79	.807
ALT (IU/L)	43.80 ± 38.37	20.17 ± 12.01	<.001
AST (IU/L)	30.70 ± 22.82	20.50 ± 5.90	<.001
GGT (IU/L)	44.94 ± 31.53	18.77 ± 12.77	<.001
ALP (IU/L)	71.78 ± 23.94	58.50 ± 15.01	<.001
TG (mmol/L)	1.71 ± 1.01	1.41 ± 0.70	.021
TC (mmol/L)	5.10 ± 0.90	4.74 ± 0.99	.018
HDL-C (mmol/L)	1.23 ± 0.42	1.41 ± 0.34	.002
LDL-C (mmol/L)	3.42 ± 0.97	3.09 ± 0.78	.016
FPG (mmol/L)	5.37 ± 0.78	4.88 ± 0.65	<.001
UA (µmol/L)	384.23 ± 85.39	303.37 ± 76.73	<.001
FIB-4	1.04 ± 0.48	0.86 ± 0.33	.003
LSM (KPa)	6.26 ± 1.88	5.21 ± 1.17	<.001

ALB = albumin, ALP = alkaline phosphatase, ALT = alanine aminotransferase, AST = aspartate aminotransferase, BMI = body mass index, FIB-4 = fibrosis-4 index, FPG = fasting plasma glucose, GGT = gamma-glutamyl transferase, HDL-C = high-density lipoprotein cholesterol, LDL-C = low-density lipoprotein cholesterol, LSM = liver stiffness measurement, MAFLD = metabolic dysfunction-associated fatty liver disease, MD = metabolic dysfunction, T2DM = type 2 diabetes mellitus, TBIL = total bilirubin, TC = total cholesterol, TG = triglyceride, UA = uric acid.

### 3.2. Serum miR-181a expression levels

**Figure 1.**
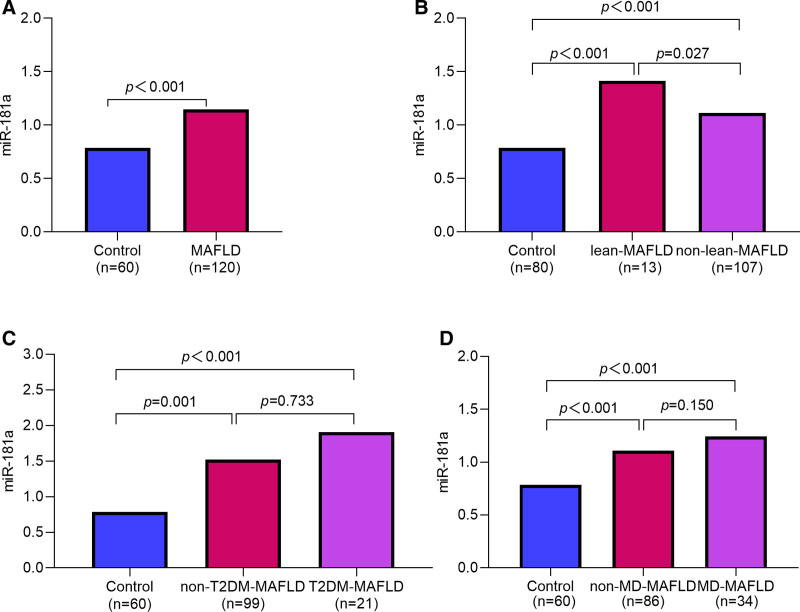
Serum levels of miR-181a. (A) All MAFLD patients versus controls. (B) MAFLD subgroups by BMI. (C) MAFLD subgroups by T2DM status. (D) MAFLD subgroups by MD status. BMI = body mass index, MAFLD = metabolic dysfunction-associated fatty liver disease, MD = metabolic dysfunction, T2DM = type 2 diabetes mellitus.

Serum miR-181a levels were significantly elevated in the MAFLD group compared to the control group (1.15 ± 0.35 vs 0.79 ± 0.23, *P* < .001) (Fig. 1A). MAFLD patients were stratified into lean (BMI < 23 kg/m²) and non-lean (BMI ≥ 23 kg/m²) subgroups. Both subgroups demonstrated significantly elevated miR-181a levels vs controls (both *P *< .001), with higher levels observed in lean vs non-lean patients (*P *= .027) (Fig. 1B). When stratified by T2DM status (non-T2DM and T2DM subgroups), both groups maintained elevated miR-181a levels relative to controls (both *P *< .001), though intergroup differences were nonsignificant (*P* = .733) (Fig. 1C). Similarly, MD-status classification (MD and non-MD subgroups) revealed elevated miR-181a levels in both groups compared to controls (both *P *< .001), without significant intergroup variation (*P* = .150) (Fig. 1D).

### 3.3. Correlation between serum miR-181a and metabolic indicators

Spearman analysis revealed that serum miR-181a levels showed significant positive correlations with several metabolic indicators, including BMI (*r* = 0.275, weak), TG (*r* = 0.222, weak), TC (*r* = 0.314, moderate), LDL-C (*r* = 0.282, weak), fasting blood glucose (*r* = 0.343, moderate), and UA (*r* = 0.246, weak) (all *P* < .05). A significant negative correlation was observed with HDL-C (r = −0.289, weak) (*P* < .001) (Table 2).

**Table 2:** Correlation of serum miR-181a with metabolic, liver function and fibrosis indicators.

Variables	*r* value	*P* value	Strength of correlation
Metabolic indicators			
BMI	0.275	<.001	Weak
TG	0.222	.0035	Weak
TC	0.314	<.001	Moderate
HDL-C	−0.289	<.001	Weak
LDL-C	0.282	<.001	Weak
FBG	0.343	<.001	Moderate
UA	0.246	.001	Weak
Liver function indicators			
ALB	0.074	.322	NS
TBIL	0.009	.903	NS
ALT	0.344	<.001	Moderate
AST	0.294	<.001	Weak
GGT	0.347	<.001	Moderate
Fibrosis indicators			
FIB-4	0.303	<0.001	Moderate
LSM	0.237	.001	Weak

Strength of spearman correlation was interpreted as follows: *r* < 0.3, weak; 0.3 ≤ *r* < 0.7, moderate; *r* ≥ 0.7, strong.

NS = not significant (*P* > .05).

ALB = albumin, ALT = alanine aminotransferase, AST = aspartate aminotransferase, BMI = body mass index, FBG = fasting blood glucose, FIB-4 = fibrosis-4 index, GGT = gamma-glutamyl transferase, HDL-C = high density lipoprotein cholesterol, LDL-C = low density lipoprotein cholesterol, LSM = liver stiffness measurement, miR-181a = microRNA-181a, TBIL = total bilirubin, TC = total cholesterol, TG = triglyceride, UA = uric acid.

### 3.4. Correlation between serum miR-181 and liver function and fibrosis

Spearman correlation analysis showed that serum miR-181a was positively correlated with liver function markers, demonstrating a moderate correlation with ALT (*r* = 0.344) and GGT (*r* = 0.347), and a weak correlation with AST (*r* = 0.294) (all *P* < .05), but not with ALB or TBIL (all *P* > .05). It also showed a moderate positive correlation with the fibrosis indicator FIB-4 (*r* = 0.303) and a weak positive correlation with LSM (*r* = 0.237) (all *P* < .001) (Table 2).

### 3.5. Logistic regression analysis of factors influencing MAFLD

Univariate logistic regression analysis identified miR-181a, BMI, T2DM, TG, TC, HDL-C, and LDL-C as significant associated factors with MAFLD (all *P* < .05). Multivariate analysis further confirmed that miR-181a, T2DM, BMI, and TG were independent predictors for MAFLD (*P* < .05) (Table 3).

**Table 3:** Logistic regression analysis of factors influencing MAFLD

Variables	Univariate		Multivariate	
	OR (95% CI)	*P* value	OR (95% CI)	*P* value
miR-181a	2.573 (1.573–9.793)	<.001	2.295 (1.318–6.236)	.011
BMI	1.626 (1.364–2.938)	<.001	1.574 (1.304–1.900)	<.001
T2DM	2.970 (1.971–9.087)	.001	2.271 (1.647–7.966)	.002
TG	1.507 (1.008–2.251)	.004	1.110 (1.167–1.836)	.006
TC	1.538 (1.084–2.183)	.016	1.201 (0.679–2.213)	.153
HDL-C	0.314 (0.140–0.705)	.005	NA*	
LDL-C	1.534 (1.050–2.241)	.027	NA*	
UA	1.013 (1.008–1.718)	.007	1.095 (0.592–1.662)	.172

BMI = body mass index, HDL-C = high density lipoprotein cholesterol, LDL-C = low density lipoprotein cholesterol, MAFLD = metabolic dysfunction-associated fatty liver disease, miR-181a = microRNA-181a, NA = not available, T2DM = type 2 diabetes mellitus, TC = total cholesterol, TG = triglyceride, UA = uric acid.

* TC comprises HDL-C and LDL-C.

### 3.6. Diagnostic value of serum miR-181a for MAFLD

**Figure 2.**
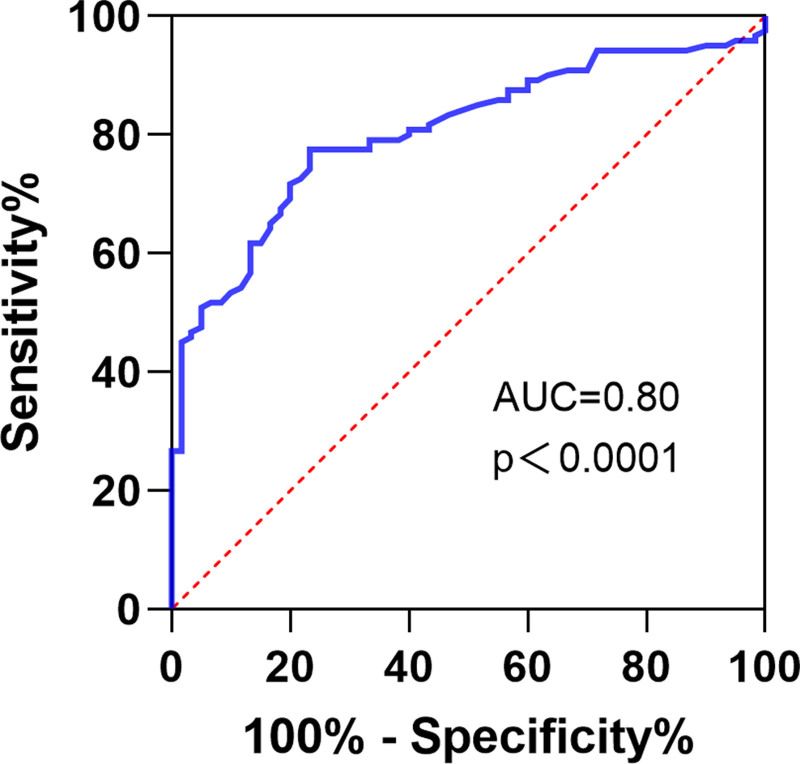
Diagnostic value of serum miR-181a for MAFLD. miR-181a = microRNA-181a, MAFLD = metabolic dysfunction-associated fatty liver disease.

ROC curve analysis demonstrated that the optimal cutoff value of serum miR-181a for the diagnosis of MAFLD was 0.90. The analysis yielded an area under the curve of 0.80, a sensitivity of 77.50%, a specificity of 76.67%, a Youden index of 54.17, a positive predictive value of 86.92%, and a negative predictive value of 63.01% (Fig. 2).

## 4. Discussion

This study demonstrates that serum levels of miR-181a are significantly elevated in patients with MAFLD and possess promising diagnostic value. We found that higher miR-181a expression was closely associated with adverse metabolic parameters, liver injury, and fibrosis, and was established as an independent risk factor for the presence of MAFLD. Given the current lack of specific serological biomarkers for MAFLD, our findings highlight serum miR-181a as a promising novel candidate for clinical diagnosis.

Given the escalating global prevalence of MAFLD, the resultant public health burden has become increasingly substantial. The pathogenesis of MAFLD entails a multifactorial framework, characterized by intricate interplay among genetic predisposition, environmental exposures, and metabolic disturbances, wherein dysregulated lipid metabolism and insulin resistance constitute pivotal pathogenic mechanisms.^[8]^ The current diagnosis of MAFLD primarily depends on imaging techniques, histological biopsy, or a combination of serum biomarkers; however, existing noninvasive biomarkers exhibit limitations in diagnostic accuracy.^[9]^ Serum biomarkers capable of reflecting the entire disease continuum of MAFLD, ranging from hepatic steatosis to fibrosis, need to be developed, particularly for differentiating simple steatosis from metabolic-associated steatohepatitis.^[10,11]^ MiRNAs, noncoding RNA molecules approximately 21 to 25 nucleotides in length, regulate essential physiological processes including adipocyte proliferation and differentiation, primarily through posttranscriptional gene silencing by targeting mRNA transcripts for degradation or translational repression.^[12]^ As a multifunctional miRNA, miR-181a participates in a wide range of biological processes, including cell proliferation, apoptosis, and invasion.^[13]^ Recent studies have demonstrated that miR-181a participates in processes related to lipid biosynthesis, lipid metabolism, and the development of obesity.^[14]^

Our study links elevated serum miR-181a to the core features of MAFLD. We found that miR-181a levels correlated with adverse lipid profiles (elevated TG, TC, LDL-C, and reduced HDL-C), liver injury markers (ALT, AST), and fibrosis indices (FIB-4, LSM), suggesting its involvement in both the metabolic dysfunction and progression of the disease. These clinical findings validate prior experimental mechanisms: The association with steatosis and dyslipidemia is explained by miR-181a-mediated downregulation of PPARα,^[6,15]^ while its link to fibrosis aligns with its role in activating hepatic stellate cells.^[7,16]^ Thus, miR-181a emerges as a key molecule connecting MD, liver damage, and fibrogenesis in MAFLD.

MAFLD typically presents without specific clinical symptoms. Although multiple noninvasive diagnostic tools are available for MAFLD screening, each exhibits certain limitations in terms of accuracy, sensitivity, or clinical applicability.^[17,18]^ Therefore, an increasing number of studies are exploring noninvasive, practical, and reliable methods for predicting MAFLD, which is also an urgent problem to be solved in clinical practice. Research shows that serum markers such as adipocyte-secreted adiponectin, leptin, and macrophage scavenger receptor 1, especially adiponectin and leptin, can participate in the occurrence and development of MAFLD by regulating liver fat accumulation, insulin resistance, and fibrosis.^[19–21]^ Some studies have also found that clinical indicators such as BMI, waist circumference, visceral fat index, lipid accumulation product, and TG-glucose index are important indicators for identifying the risk of MAFLD.^[22–25]^ In this study, the diagnostic performance of miR-181a in our ROC analysis underscores its potential for clinical translation. This suggests that a standardized miR-181a assay could be deployed as a screening tool for MAFLD in primary care settings, particularly for high-risk individuals. Moreover, its significant correlation with fibrosis markers indicates an additional role in risk stratification, potentially helping to identify patients with progressive disease who warrant closer monitoring. Future efforts to develop a cost-effective assay and define clinically validated cutoff values are essential next steps.

This study has several limitations. 1st, this study has a single-center design, which may limit the generalizability of our findings. Therefore, external validation through multicenter studies with larger and more diverse populations is necessary. 2nd, the mechanisms of miR-181a in MAFLD remain unclear and require further experimental study. 3rd, liver fibrosis was assessed using only noninvasive markers, without histological confirmation by liver biopsy in any participants.

In conclusion, miR-181a is significantly associated with the pathogenesis and progression of MAFLD and shows promise as a diagnostic biomarker for this condition. However, the underlying mechanisms of miR-181a in MAFLD remain to be elucidated.

## Author contributions

**Data curation:** Kun-Yu Ke, Ke-Gong Xiong.

**Supervision:** Kun-Yu Ke.

**Writing – review & editing:** Kun-Yu Ke.

**Conceptualization:** Ke-Gong Xiong.

**Formal analysis:** Ke-Gong Xiong, Jin-Feng Kong, Tai-Shun Lin.

**Funding acquisition:** Ke-Gong Xiong.

**Investigation:** Ke-Gong Xiong, Jin-Feng Kong, Tai-Shun Lin, Qing-Biao Lin.

**Writing – original draft:** Ke-Gong Xiong.

**Validation:** Jin-Feng Kong.

## References

[1] Eslam M Newsome PN Sarin SK. A new definition for metabolic dysfunction-associated fatty liver disease: an international expert consensus statement. J Hepatol. 2020;73:202–9. 32278004 10.1016/j.jhep.2020.03.039

[2] Eslam M Fan JG Yu ML. The Asian Pacific association for the study of the liver clinical practice guidelines for the diagnosis and management of metabolic dysfunction-associated fatty liver disease. Hepatol Int. 2025;19:261–301. 40016576 10.1007/s12072-024-10774-3

[3] Feng G Targher G Byrne CD. Global burden of metabolic dysfunction-associated steatotic liver disease, 2010 to 2021. JHEP Rep. 2025;7:101271. 39980749 10.1016/j.jhepr.2024.101271 PMC11840544

[4] Gierlikowski W Gierlikowska B. MicroRNAs as regulators of phagocytosis. Cells. 2022;11:1380. 35563685 10.3390/cells11091380 PMC9106007

[5] Zhou B Li C Qi W. Downregulation of miR-181a upregulates sirtuin-1 (SIRT1) and improves hepatic insulin sensitivity. Diabetologia. 2012;55:2032–43. 22476949 10.1007/s00125-012-2539-8

[6] Huang R Duan X Liu X. Upregulation of miR-181a impairs lipid metabolism by targeting PPARα expression in nonalcoholic fatty liver disease. Biochem Biophys Res Commun. 2019;508:1252–8. 30558790 10.1016/j.bbrc.2018.12.061

[7] Wang Y Mou Q Zhu Z Zhao L Zhu L. MALAT1 promotes liver fibrosis by sponging miR‑181a and activating TLR4‑NF‑κB signaling. Int J Mol Med. 2021;48:215. 34651657 10.3892/ijmm.2021.5048 PMC8547543

[8] Portincasa P Khalil M Mahdi L. Metabolic dysfunction–associated steatotic liver disease: from pathogenesis to current therapeutic options. Int J Mol Sci . 2024;25:5640. 38891828 10.3390/ijms25115640 PMC11172019

[9] Hou M Gu Q Cui J. Proportion and clinical characteristics of metabolic-associated fatty liver disease and associated liver fibrosis in an urban Chinese population. Chin Med J (Engl). 2024;138:829–37. 39183555 10.1097/CM9.0000000000003141 PMC11970824

[10] Keating SE Sabag A Hallsworth K. Exercise in the management of metabolic-associated fatty liver disease (MAFLD) in adults: a position statement from exercise and sport science Australia. Sports Med. 2023;53:2347–71. 37695493 10.1007/s40279-023-01918-w PMC10687186

[11] Cheng PN Chen WJ Hou CY. Taiwan association for the study of the liver-taiwan society of cardiology Taiwan position statement for the management of metabolic dysfunction- associated fatty liver disease and cardiovascular diseases. Clin Mol Hepatol. 2024;30:16–36. 37793641 10.3350/cmh.2023.0315 PMC10776290

[12] Doghish AS Mansour RM Abdel Mageed SS. Role and significance of MicroRNAs in the relationship between obesity and cancer. Balkan Med J. 2025;42:188–200. 40326803 10.4274/balkanmedj.galenos.2025.2025-3-60 PMC12060578

[13] Florian IA Buruiana A Timis TL. An insight into the microRNAs associated with arteriovenous and cavernous malformations of the brain. Cells. 2021;10:1373. 34199498 10.3390/cells10061373 PMC8227573

[14] Hongfang G Khan R El-Mansi AA. Bioinformatics analysis of miR-181a and its role in adipogenesis, obesity, and lipid metabolism through review of literature. Mol Biotechnol. 2023;66:2710–24. 37773313 10.1007/s12033-023-00894-w

[15] Wang T Liu Y Wu X. Multi-omics reveals miR-181a-5p regulates PPAR-driven lipid metabolism in oral squamous cell carcinoma: insights from CRISPR/Cas9 knockout models. J Proteomics. 2025;319:105480. 40490244 10.1016/j.jprot.2025.105480

[16] Dong Z Yang X Qiu T. Exosomal miR-181a-2-3p derived from citreoviridin-treated hepatocytes activates hepatic stellate cells trough inducing mitochondrial calcium overload. Chem Biol Interact. 2022;358:109899. 35305974 10.1016/j.cbi.2022.109899

[17] Pal SC Méndez-Sánchez N. Screening for MAFLD: who, when and how? Ther Adv Endocrinol Metab. 2023;14:20420188221145650. 36699945 10.1177/20420188221145650 PMC9869195

[18] Qu B Li Z. Exploring non-invasive diagnostics for metabolic dysfunction-associated fatty liver disease. World J Gastroenterol. 2024;30:3447–51. 39091712 10.3748/wjg.v30.i28.3447 PMC11290396

[19] Kim KE Lee J Shin HJ. Lipocalin‐2 activates hepatic stellate cells and promotes nonalcoholic steatohepatitis in high‐fat diet–fed Ob/Ob mice. Hepatology. 2023;77:888–901. 35560370 10.1002/hep.32569 PMC9936980

[20] Pal SC Eslam M Mendez-Sanchez N. Detangling the interrelations between MAFLD, insulin resistance, and key hormones. Hormones (Athens). 2022;21:573–89. 35921046 10.1007/s42000-022-00391-w

[21] Govaere O Petersen SK Martinez-Lopez N. Macrophage scavenger receptor 1 mediates lipid-induced inflammation in non-alcoholic fatty liver disease. J Hepatol. 2022;76:1001–12. 34942286 10.1016/j.jhep.2021.12.012 PMC7619241

[22] Peng H Pan L Ran S. Prediction of MAFLD and NAFLD using different screening indexes: a cross-sectional study in U.S. adults. Front Endocrinol. 2023;14:1083032. 10.3389/fendo.2023.1083032 PMC9892768 36742412

[23] Wang H Zhang Y Liu Y. Comparison between traditional and new obesity measurement index for screening metabolic associated fatty liver disease. Front Endocrinol. 2023;14:1163682. 10.3389/fendo.2023.1163682 PMC10160459 37152940

[24] Liu J Duan S Wang C. Optimum non-invasive predictive indicators for metabolic dysfunction-associated fatty liver disease and its subgroups in the Chinese population: a retrospective case-control study. Front Endocrinol. 2022;13:1035418. 10.3389/fendo.2022.1035418 PMC9751395 36531447

[25] Lv D Wang Z Liu H Meng C. Predictive value of the triglyceride-glucose index for metabolic-associated fatty liver disease in individuals with different metabolic obese phenotypes. Diabetes Metab Syndr Obes: Targets Ther. 2025;18:125–33. 10.2147/DMSO.S500042 PMC11742748 39834613

